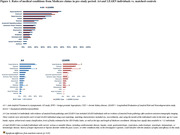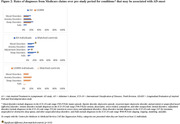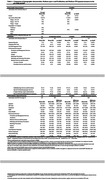# Healthcare resource utilization in cognitively unimpaired individuals 5 years prior to amyloid PET for A4 vs. LEARN eligibility

**DOI:** 10.1002/alz.087765

**Published:** 2025-01-09

**Authors:** Julie Chandler, Joanna Kubisiak, Angelina Lee, Karen Chilcott Holdridge, Rebecca L. Robinson, Roy Yaari, Michael Rafii, Paul S. S. Aisen, Reisa A Sperling

**Affiliations:** ^1^ Eli Lilly and Company, Indianapolis, IN USA; ^2^ Westat Inc., Rockville, MD USA; ^3^ University of Southern California, San Diego, CA USA; ^4^ Center for Alzheimer’s Research and Treatment, Brigham and Women’s Hospital/Harvard Medical School, Boston, MA USA

## Abstract

**Background:**

Medical history and healthcare utilization in preclinical Alzheimer’s disease (AD) are not well characterized and may reveal indicators associated with asymptomatic stages of AD.

**Methods:**

This retrospective observational study compared 246 Anti‐Amyloid Treatment in Asymptomatic AD study (A4) individuals who met elevated brain amyloid eligibility criteria to 121 individuals in the companion Longitudinal Evaluation of Amyloid Risk and Neurodegeneration study (LEARN) who were eligible for A4 except did not meet elevated amyloid eligibility criteria. Matched‐controls for A4/LEARN, using a 3:1 match of demographics, Medicare enrollment month, and frailty status, were randomly selected from Medicare beneficiaries without cognitive impairment/dementia claims. Primary outcomes (Medicare claims diagnoses, utilization, payments, medications) were compared up to 5 years before A4/LEARN enrollment.

**Results:**

A4 and LEARN individuals had comparable common comorbidities during 5‐year pre‐study period; hypertension and diabetes were significantly less frequent vs. matched‐controls (**Figure 1**). Rates of diagnoses associated with AD onset (mood, adjustment, sleep disorders), were generally similar between A4 and LEARN, but significantly higher among A4 but not LEARN individuals vs. matched‐controls (**Figure 2**).

Utilization for inpatient, emergency room, professional encounters for evaluation/management, surgery, anesthesia, and pathology/laboratory services were significantly higher for A4 vs. LEARN individuals (Table 1). Relative to matched‐controls, A4 and LEARN individuals had generally higher rates of professional encounters; LEARN had lower inpatient episodes.

A4 vs. LEARN individuals had higher payments for ER and anesthesia (**Table 1**). A4 individuals vs. matched‐controls had higher payments for medicine services/procedures but lower payments for durable medical equipment.

A4 individuals had similar or greater use of all medication categories except for cardiovascular relative to matched‐controls.

**Conclusions:**

Up to 5 years before amyloid testing, individuals who met elevated amyloid eligibility criteria had comorbidity profiles generally comparable to companion study individuals who did not meet amyloid eligibility criteria and matched‐controls, but were more likely than matched‐controls to have diagnoses associated with AD onset, and had greater utilization of medicine services and procedures. The latter may indicate a predisposition to seeking medical care among individuals who participate in clinical studies. Continued follow‐up of these patterns may provide additional insight into early indicators associated with asymptomatic AD.